# New synthetic route for polyricinoleic acid with Tin (II) 2-ethylhexanoate

**DOI:** 10.1016/j.heliyon.2019.e01944

**Published:** 2019-06-18

**Authors:** Rajeshkumar Natwarlal Vadgama, Annamma Anil Odaneth, Arvind Mallinath Lali

**Affiliations:** DBT-ICT Centre for Energy Biosciences, Institute of Chemical Technology, Nathalal Parikh Marg, Matunga (E), Mumbai, 400 019, India

**Keywords:** Natural product chemistry, Analytical chemistry, Polyricinoleic acid, Ricinoleic acid, Tin (II) 2-ethylhexanoate

## Abstract

Polyricinoleic acid (PRA) is a biodegradable polymer of ricinoleic acid, a hydroxy fatty acid present in castor oil. Depending upon the chain length, this homopolymer finds varied applications in oleochemical and allied industries. In the current research, we have first demonstrated synthesis route for PRA using Tin (II) 2-ethylhexanoate as a catalyst. The multiple operational variables such as the effect of the reaction time, temperature, catalyst loading, water condensation and recuperation of polymer were studied systemically. Under the optimized conditions, PRA with a molecular weight of ∼4kD was synthesized at 150 °C within 14 hrs. Using solvent extraction, the unreacted monomer and catalyst were recycled back to the next round of polymerization. The final PRA product was characterized as a single molecule with superior functional properties and suggesting to its use as an environmentally friendly biopolymer.

## 1 Introduction

Vegetable oils, a hotspot for both academic and industrial research, acts as platform chemicals for the synthesis of various polymeric materials. The components of vegetable oils have several highly reactive sites, including double bonds, allylic positions, and tester groups. These provide a great opportunity to form a variety of polymers with different structures and functionalities [1]. the development of polymer with defined structures and properties, aimed specifically at bio-based applications, propels research on the biodegradable polymer synthesis. Consequently, a biopolymer synthesis with defined structure with high functionality is an urgent need in a current scenario [2].

Bio-based polymers are considered to be potential future candidate materials for polymeric applications [3, 4]. Biopolymers such as estolides, which are oligomeric polyesters of fatty acids, can be produced chemically or biotechnologically for their incorporation in food, cosmetics, lubricants and other desirable applications [5]. The ability to modify the fatty acid functionality with esters of various chain lengths and branching, along with the potential to build up incremental molecular weights (MW) through polymerization, provides a good route to making molecules with targeted physical properties.

There are varieties of estolids prepared, including ricinoleic acid (C-18 hydroxy fatty acid) present in castor oil [6, 7]. Novel ricinoleic acid based estolides [8] and cyclic carbonate [9] have been synthesized. Further, Martin-Arjol et al reported Mono-Estolide Synthesis from trans-8-Hydroxy-Fatty Acids by Lipases in Solvent-Free Media [10]. Oleic and ricinoleic acids-derived estolides have been studied using different acid-catalysed synthesis protocols [11].

Polyricinoleic acid (PRA) made from ricinoleic acid is an estolide that has a variety of potential applications in the field of lubricants, hydraulic fluids, greases, plastics, inks, foods, cosmetics, and surfactants. These estolides are increasingly being used as a viscosity controller for chocolate, low-fat spreads and as emulsifiers in margarine, as cutting oil base in metal processing, and also as a pigment dispersal in paint, ink, and cosmetics [12].

Different applications of PRA demand polyesters with varying acid values (AV) depending on the physical properties of the final product. Applications requiring fluidity as in shampoos have requirements for shorter polyesters with an AV of 60–90 (mg KOH/g) in contrast to an AV of 20–40 (mg KOH/g) for cosmetic formulations. Thus, defined esterification is necessary for achieving PRA of desired AV and physicochemical properties [13].

PRA synthesis has been reported from castor oil [14] and the homopolymerization of ricinoleic acid by enzymatic [15, 16, 17] or chemical routes. The issues related to enzymatic routes such as high cost of catalyst, low operational stability, and incomplete conversions can be resolved using a chemical catalyst for efficient PRA synthesis. Modak and Kane's work demonstrated the formation of PRA by the homopolymerization reaction at 202 °C which gave an AV of 38 mg KOH/g Oil [18]. Based on the molecular weight of ricinoleic acid, this molecule would have contained 5 ricinoleate groups, thus estolide number (EN) = 4 with a molecular weight of 1,476 Da. However, no further study has been attempted to synthesize PRA with different molecular weights using chemical catalysts.

In the present research, we first time showed the synthesis of PRA using a chemical catalyst, Tin (II) 2-ethylhexanoate. Previously, the use of Tin (II) 2-ethylhexanoate as a catalyst in the polymerization of lactic acid, a C-3 hydroxy acid, has been reported. This widely used catalyst has high solubility in many starting reactants, low toxicity, Food and Drug Administration (FDA) approval, high catalytic activity, and ability to give high-molecular-weight polymers with low racemisation [19, 20, 21]. However, this catalyst has not been reported, to the best of our knowledge, for polymerization of long chain hydroxy fatty acid such as ricinoleic acid. Here, we are reporting a novel homopolymerization of long chain aliphatic and bi-functional ricinoleic acid using Tin (II) 2-ethylhexanoate. The new synthetic route for polymerization of ricinoleic acid is being introduced and various variables affecting polymerization reaction were systematically studied. In addition, the improvement of biopolymer property, as well as catalyst recycling using solvent extraction, was investigated.

## 2 Materials and methods

### 2.1 Materials

Ricinoleic acid (Technical grade - 89 % monomer, 11% Dimer) was obtained as a free sample from Acme Synthetic Chemical (ASC), Mumbai. Tin (II) 2-ethylhexanoate was procured from Sigma-Aldrich Ltd. Methanol, ethanol and isopropyl alcohol reagent were purchased from S.D. Fine Chemicals, India. All the other chemicals and reagent were of analytical grade.

### 2.2 Polymerization reaction

**Scheme 1:**
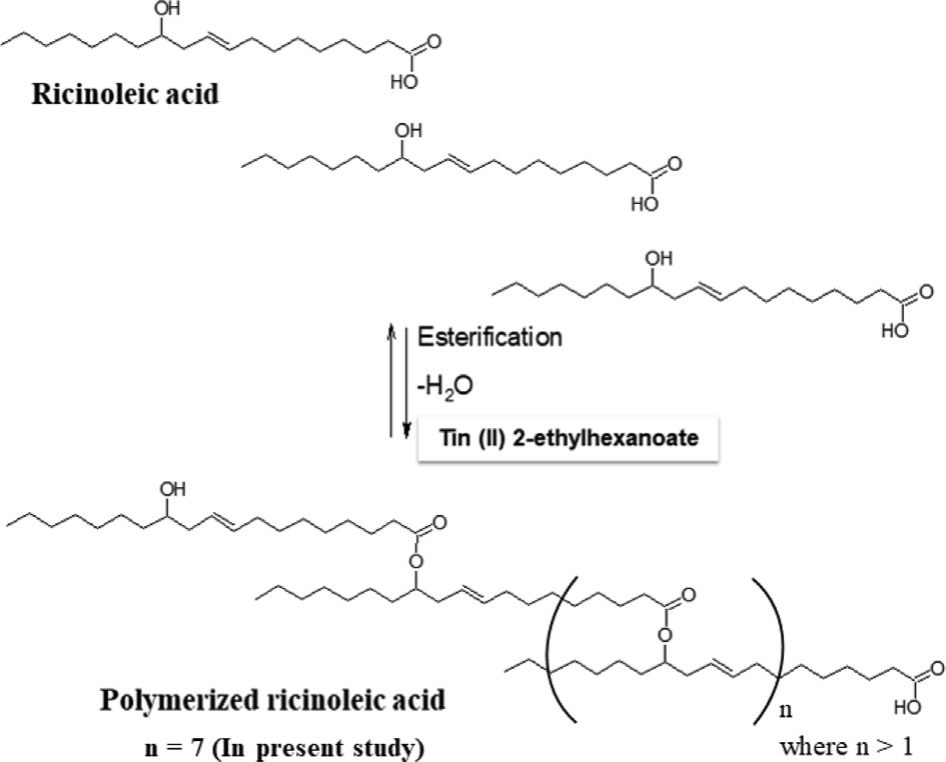
Polymerization of Ricinoleic acid.

The polymerization reaction was carried out in a STEM reactor composed of six round compartments with a magnetic stirrer and temperature control system. The reaction was started by adding catalyst (5% w/w) into ricinoleic acid (10 g) placed in the compartment of the reactor. Samples were taken at a defined time interval and analyzed for product formation. The process of polymerization is shown in Scheme 1.

### 2.3 Product analysis

#### 2.3.1 Acid value (AV)

AVs were determined using modified ASTM D 664 (2007) method performed on an Ion analysis auto-titrator (Metrohm, Switzerland). About 1 g of completely dried sample was dissolved in 25 ml solvent mixture (IPA: methanol, 1:2 v/v) which was titrated against standard 0.1 N alcoholic KOH. AV (mg KOH/g sample) was then calculated as follows:

$$\text{Acid}\;\text{value}\;\text{(AV)}\;=\;\frac{56.11\times KOH\;normality\;\times \;'V'\;ml}{Sample\;weight}$$

#### 2.3.2 GPC analysis

The Non-aqueous Gel Permeation Chromatography (GPC) analysis was done to to assess the molecular weight and molecular weight distribution of polymer. Two Agilent GPC columns, PL mixed gel E (3μ, 300 × 7.5 mm) and PL mixed gel D (5μ, 300 × 7.5 mm), were connected in series, following a guard column (5 μ, 50 × 7.5 mm) and maintained at 40 °C. The elution was done by THF with a flow rate of 1 ml/min and UV detection was set at 240 nm.

#### 2.3.3 FTIR analysis

Synthesized liquid polyricinoleic acid sample was subjected to FTIR analysis. The ester functional group in formed polymer was analyzed using Fourier Transform Infrared Spectrophotometer, IR Prestige-21 (Shimadzu, Japan) equipped with DlaTGS detector. The spectrum was recorded from co-addition of 30 scans at resolution of 4 cm^−1^.

### 2.4 Recovery of catalyst and final product

For separation of catalyst and polymer, 1 g of sample with 5 ml of respective organic solvent was taken into separating funnel (50 ml). After vigorous shaking for 5 minutes, the layers were allowed to separate. The upper soluble fraction contains catalyst and oligomers while the lower insoluble fraction contains PRA as a final product. Samples from both layers were analyzed by acid value and GPC.

## 3 Results and discussion

### 3.1 Effect of the reaction time

**Fig. 1:**
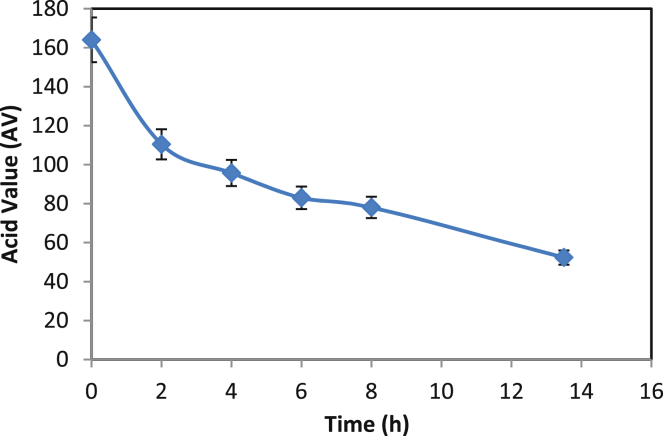
Effect of reaction time on PRA synthesis. [Catalyst cocn = 5% w/w, temperature of reaction = 150°C]

**Fig. 2:**
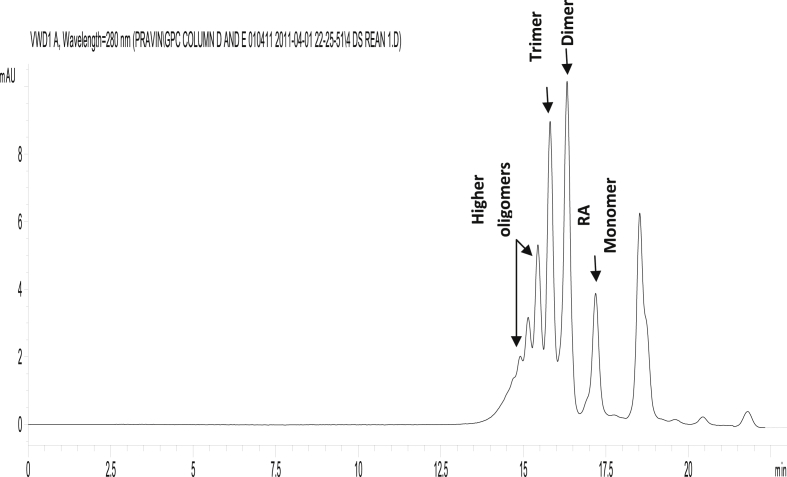
GPC profile of the synthesized PRA.

Reaction time is a good indicator of catalyst performance and reaction progress. It helps to pinpoint the adequate time necessary to obtain the best yield and minimize the process cost. Fig. 1 shows the AV profile of Tin (II) 2-ethylhexanoate catalyzed the synthesis of PRA. As the reaction progresses, the hydroxyl group of one monomer reacts with the carboxylic group of another monomer to form oligomer and then the chain was lengthened to form a polymer. This was indicated by a decrease in the AV from 164 to 52 (mg of KOH/g sample). The GPC profile of the synthesized PRA (AV = 52) is shown in Fig. 2. It was demonstrated for the first time that Tin (II) 2-ethylhexanoate can catalyze the polymerization of ricinoleic acid. Earlier, Raia Slivniak *et al* reported the polymerization of the ricinoleic acid lactones using Tin (II) 2-ethylhexanoate a catalyst [22]. The various catalysts were tried for PRA synthesis (see Table 1), but Tin (II) 2-ethylhexanoate has given better results and thus the reaction parameters such as temperature, catalyst concentrations, etc. were further investigated with Tin (II) 2-ethylhexanoate as catalyst for PRA synthesis.

**Table 1:** Comparative analyses with alternative catalysts for PRA synthesis.

Catalyst	Time Peroid (h)	Temperaure (°C)	Product MW range (Da)
Immbilized Lipase	72	60	298–858
Ion exchange resin	48	95	298–1400
Tin (II) 2-ethylhexanoate (Present study)	14	150	1018–4000

### 3.2 Effect of temperature

**Fig. 3:**
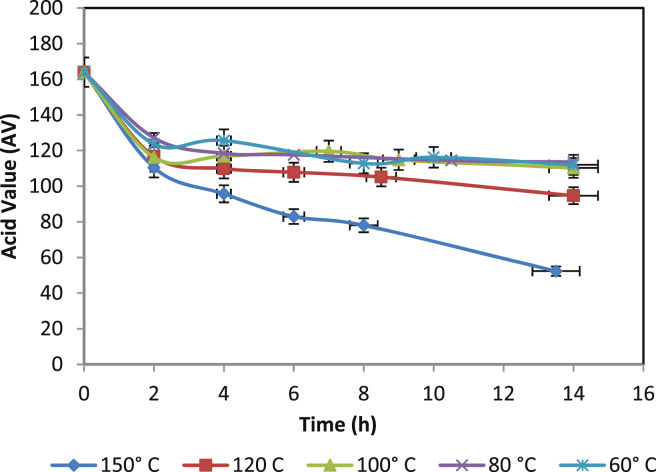
Effect of temperature on PRA synthesis [Catalyst cocn = 5% w/w/].

Reaction temperature is a crucial parameter in catalysis [23], therefore, we have selected five different temperatures in the range of 60–150 °C for the synthesis of PRA (Fig. 3). It was observed that the increase in temperature accelerating PRA synthesis. Ordinarily, it is related to an increase in the kinetic energy of the system as well as collisions between a catalyst and substrate molecules. Moreover, it was observed that temperature above 120 °C favoured PRA synthesis due to the evaporation of water (by-product) which shifts the equilibrium towards the synthesis side. Previously, Buasri *et al* reported 150–160 °C temperature for the synthesis of PET-PLA Co-polyester using Tin (II) 2-ethylhexanoate as a catalyst [24].

### 3.3 Effect of catalyst concentration

**Fig. 4:**
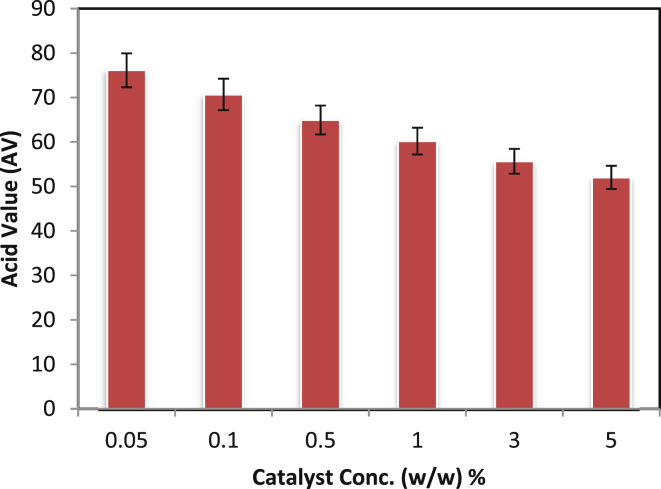
Effect of catalyst concentration on PRA synthesis [Temperature of reaction = 150°C, time of reaction = 14 hrs].

The amount of catalyst used should be as low as possible to obtain the desired product. This is because it adds to the process cost and hence to the industrial acceptance for large scale production. Catalyst concentration influences the rate of reaction and therefore it is assessed to facilitate determination of the minimal amount necessary for achieving a good yield. The influence of varying Tin (II) 2-ethylhexanoate concentration from 1 - 5% (w/w) was studied (Fig. 4). As the catalyst concentration increased, PRA with lower AV was formed in given the time period. A critical amount of catalyst is necessary for equilibrium amongst the reactant and product in the system [25, 26]. Maximum polymerizations (AV = 52) was observed with 5% catalyst and hence it was used for further studies.

### 3.4 Effect of water condensation

**Fig. 5:**
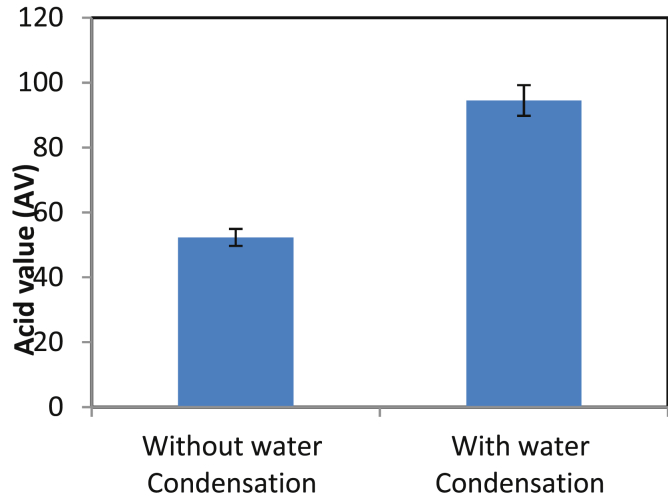
Effect of water condensation on PRA synthesis[Catalyst Concn = 5% w/w, Temperature of reaction = 150°C, time of reaction = 14 hrs].

In esterification reactions, the water produced in the reaction medium can negatively affect the equilibrium as well as the distribution of products in medium [27]. Therefore, optimization of the water present during polyesterification is important. It is known that Tin (II) 2-ethylhexanoate shows lower sensitivity towards the water and other impurities [28]. To determine the influence of water on the polyesterification reaction, an experiment was carried out in two types of reactor i.e. open and closed. When the polymerization reaction was conducted in a closed container, the sustainable increase in AV (AV of 100 vs. 52) was observed (Fig. 5). The water generated during polymerization gets condensed and subsequently shifts the equilibrium towards hydrolysis. It could be inferred that chemical catalyst, Tin (II) 2-ethylhexanoate, catalyzes polyesterification reaction in both directions depending upon the microenvironment conditions.

### 3.5 Screening of organic solvent

The PRA product formed under optimized reaction is a mixture of unreacted monomer, oligomers, and polymer along with the catalyst. This can be seen from the data presented in Table 2. The polydispersity index is more than one which implicates that it is the mixture of unreacted monomer, oligomers, and polymer.

**Table 2:** Characterization of polyricinoleic acid.

Property of PRA product	Value
Number Average Molecular Weight (M_n_)	2158 Da
Weight Average Molecular Weight (M_w_)	2195 Da
Polydispersity index (PDI)	1.017
Degree of Polymerization (X_n_)	7.24

**Fig. 6:**
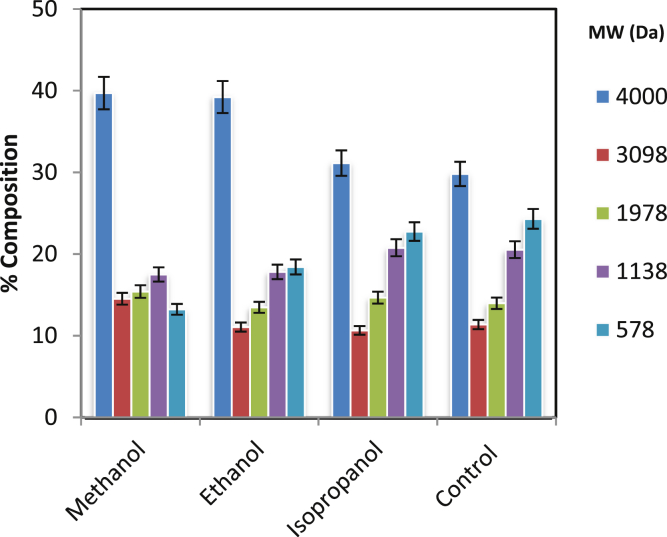
GPC analysis of insoluble fraction.

**Fig. 7:**
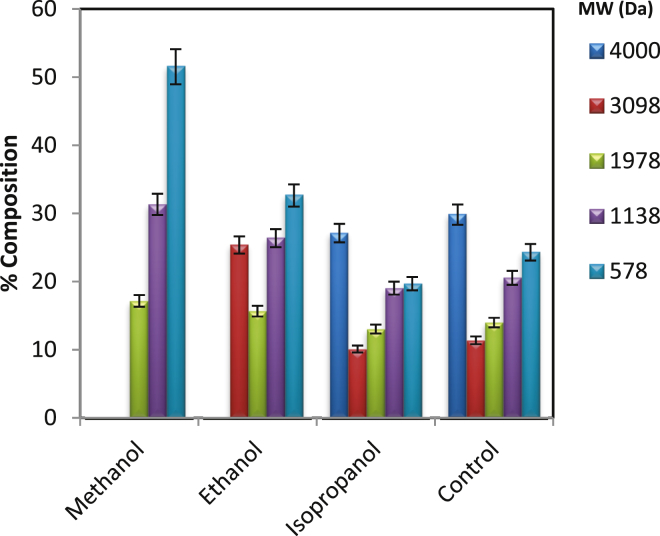
GPC analysis of soluble fraction.

To separate catalyst or unreacted monomer or oligomers from polymer, the attempt was made using organic solvent based on the difference in physical properties of molecules. The screening of different polar organic solvents viz. Methanol, Ethanol and Isopropyl alcohol were carried out. The separation was observed because of different solubility of catalyst/monomer/oligomers and polymer. There was the formation of two distinct layers on the addition of solvent into PRA product. These layers, i.e. upper (soluble fraction) and lower (insoluble fraction) were collected separately and analyzed by GPC for molecular weight determination (Figs. 6 and 7). It can be seen that methanol, being most polar, selectively extracts low MW oligomer and catalyst leaving behind PRA with MW > 3000 Da. This could be attributed according to the log P values of solvent used, i.e. Log P value of Isopropanol (0.05) > Ethanol (−0.3) > Methanol (−0.74). Thus, the polarity of solvent play a decisive role in polymer and monomer separation. The catalyst, which is highly soluble in methanol, was separated from high MW PRA. The initial concentration of catalyst (as Tin) was 200 ppm which was then found to be 2 ppm in the final product after washing with methanol. Separated low MW oligomer and catalyst can be recycled back to PRA synthesis.

### 3.6 Optimization of solvent to product ratio

**Fig. 8:**
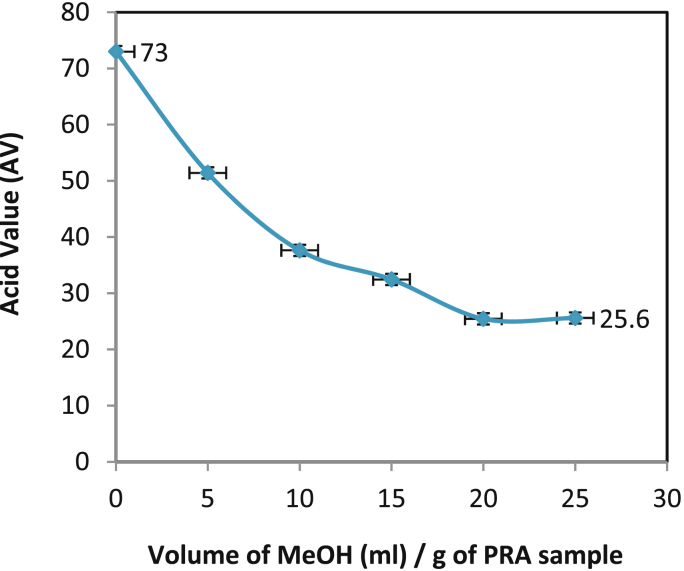
Optimization of methanol to PRA ratio.

The optimization of PRA to methanol ratio was performed to obtain a homogenous polymer with high MW. As amounts of methanol increased, the AV of PRA product decreased i.e. purity increased (Fig. 8). After 20 ml of methanol per g of PRA product, no significant change in AV (25 mg/g of KOH) was seen and GPC analysis showed that the final PRA product (insoluble fraction) was homogenous with high MW (4026 ± 440 Da). All other molecules with MW < 4026 were solubilized and entered into an upper layer of methanol. Thus, an optimized ratio of methanol (20 ml/g of sample) to PRA yields polymer with high MW. The yield of this product was ∼47 % based on starting monomer. The final product characterization profile is presented in Table 3.

**Table 3:** Characterization of final polyricinoleic acid product.

Property of PRA product	Value
Molecular Weight (MW)	4026 ± 440 Da
Acid Value (mg KOH/g)	25.6
Hydroxyl value (mg KOH/g)	12.3
Estolide Number (EN)	13
Gardner colour scale	6
Viscosity (at 25 °C)	1200 cSt

### 3.7 FTIR analysis of final polyricinoleic acid product

**Fig. 9:**
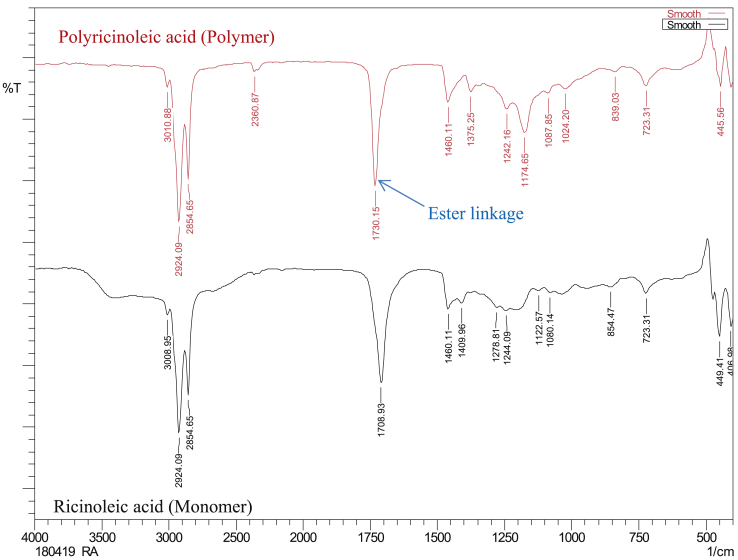
FTIR analysis of final polyricinoleic acid product.

The FTIR spectrum of the final polyricinoleic acid product is shown in Fig. 9. The band at 3010.88 cm^−1^ is due to the hydroxyl stretching, band in the region of 2924.09 cm^−1^ and 2854.65 cm^−1^ is due to C–H stretching of –CH_2_. The characteristic band in region of 1730.15 cm^−1^ is due to C=O stretching of ester synthesized. Also the absorption in the regions 1174.86 cm^−1^ is due to C–O–C bond in esterified ricinoleisc acid.

## 4 Conclusion

The polyesterification of ricinoleic acid had been carried out successfully using Tin (II) 2-ethylhexanoate as a catalyst. Polymerization reaction affecting variables like reaction time, temperature, catalyst loading, water condensation, types of reactors were investigated. Further, polarity and the amount of organic solvent decided the fate for obtaining PRA with high purity and MW. The final PRA product so obtained was characterized as a single molecule (4026 ± 440 Da) by GPC analysis and its physical properties suggests its use as an eco-friendly polymeric material. A unique method for production of PRA from ricinoleic acid using Tin (II) 2-ethylhexanoate as catalyst followed by solvent extraction could be proposed.

## Declarations

### Author contribution statement

Rajeshkumar Vadgama: Conceived and designed the experiments; Performed the experiments; Analyzed and interpreted the data; Wrote the paper.

Annamma Anil: Conceived and designed the experiments; Analyzed and interpreted the data.

Arvind Lali: Conceived and designed the experiments; Contributed reagents, materials, analysis tools or data.

### Funding statement

This research did not receive any specific grant from funding agencies in the public, commercial, or not-for-profit sectors.

### Competing interest statement

The authors declare no conflict of interest.

### Additional information

No additional information is available for this paper.
